# Robotic vs. TaTME Rectal Surgery (ROTA STUDY) Matched Cohort Trial for Mid to Low Rectal Cancer Surgery Evaluation Trial in the Hands of an Experienced Surgeon

**DOI:** 10.29337/ijsp.163

**Published:** 2022-02-18

**Authors:** Ravish Jootun, Pedja Cuk, Mark Ellebæk, Per Vadgaard Andersen, Søren Salomon, Gunnar Baatrup, Issam Al-Najami, Jim Khan

**Affiliations:** 1 Department of Colorectal Surgery, Austin hospital, Heidelberg, Victoria, Australia; 2 Department of Surgery, Hospital of Southern Jutland, Denmark; 3 Department of regional health research, University of Southern Denmark, Denmark; 4 Department of Surgery Odense University Hospital, Svendborg, Denmark; 5 Department of Colorectal Surgery Queen Alexandra Hospital, Portsmouth, UK

**Keywords:** Rectal cancer, Total mesorectal excision, Robotic surgery, trans-anal surgery

## Abstract

**Background:**

Recent novel surgical techniques for resection of low rectal cancer have been introduced and these approaches have the potential to overcome anatomical limitations like obesity, narrow male pelvis and bulky and low tumours. Two of these procedures are robotic low anterior resection (RLAR) and transanal total mesorectal excision (TaTME).

Both approaches have distinct advantages and limitations. There has been no head to head trial comparing RLAR and TaTME for patients with mid to low rectal cancer undergoing surgery by experienced surgeons. Previous studies looking at the oncological outcomes of either TaTME or robotic TME included many centres where the surgeons were on a learning curve and hence the true oncological outcomes and clinical benefits can not be measured accurately.

**Method:**

The inclusion criteria include experienced surgeons defined as minimum of 60 prior procedures with RLAR or TaTME. Successful oncological and clinical outcomes are defined as circumferential resection margin (CRM) ≥1 mm with limited postoperative morbidity (absence of Clavien-Dindo grade III–IV complications within 30 days after surgery). Local and distal recurrence rates with DFS over 3 years will be measured as primary outcome.

Data will be collected prospectively and entered in a dedicated database.

**Discussion:**

The primary objective of this study is to conduct a multicentre prospective trial to investigate clinical outcomes, in particular disease free survival (DFS) in patients undergoing RLAR and TaTME. The additional goal is to investigate other efficacy measures, complications rates, health economic aspects and patient reported health related quality of life.

This paper describes an important trial conducted in expert centres to establish the needed knowledge for a detailed comparison of outcomes for TaTME versus RLAR.

This trial is the first comparative study, comparing TaTME and RLAR, seeking to establish foothold for tailor-made surgical treatment of low rectal cancer patients.

**Trial registration:**

The trial is registered in *clinicaltrials.gov* September 2019. *Clinicaltrials.gov* id: NCT04200027.

## Background

The outcome of surgery for rectal cancer has improved substantially during the past two decades due to the introduction of the TME technique, which involves complete removal of the mesorectum with preservation of the pelvic autonomic nerves. Local recurrence rate of rectal cancer has been reduced [1] because discontinuous cancer cells in the mesorectum are removed by complete resection of this tissue [1,2,3].

Minimally invasive surgery is taking over as the preferred approach for colorectal diseases. Recently published randomized clinical trials (RCTs), such as COLOR II, COREAN and CLASICC, have shown better results for laparoscopic total mesorectal excision in terms of short-term and long-term outcomes, when compared with open TME [4,5,6].

Sphincter saving TME surgery of ultra low rectal cancer is technically demanding and hold a higher risk of incomplete resection. It is offered to a small fraction of patients in few dedicated centres. TME is limited in patients with low rectal cancer, who require surgeons with experience in ultra-low sphincter-saving laparoscopic surgery, which has a high risk of leaving a positive circumferential resection margin (CRM). In addition, narrow pelvic anatomy; male sex and high body mass index (BMI) are also unfavourable patient characteristics for a laparoscopic approach.

The wish to overcome these challenges has motivated surgeons to explore alternative techniques. The two more recent procedures that have been introduced are robotic low anterior resection (RLAR) and Transanal Total Mesorectal Excision (TaTME). These two new procedures have the potential to overcome anatomical limitations like obesity, narrow male pelvis and bulky and low tumours.

Transanal assissted TME (TaTME) combines the TAMIS (transanal minimally invasive surgery) and trans-abdominal approaches to successfully perform the TME. TaTME typically starts low in the pelvis and progresses to the mobilization of the splenic flexure/sigmoid colon in a “bottom-up” manner [7]. A single-incision laparoscopic surgery port is introduced into the anal canal to gain endoscopic access to the rectum, pneumorectum is established, A purse-string suture is applied below the tumor to ensure an adequate distal margin [8]. Transanal excision is subsequently performed with laparoscopic instruments.

Rectal distension with CO_2_ combined with magnifying optics permits excellent visualization of tissue planes. Easier access to the low rectum and precise selection of the distal resection margin under direct visualization helps ensure an adequate margin. It has been argued that TaTME may lead to a safer anastomosis by avoiding the multiple stapler firings often required in the laparoscopic approach and may result in higher rates of sphincter preserving surgery.

The robotic approach has been subject to much interest in recent years because of its potential learning benefits and clinical benefits to the patients. Some of the clinical benefits include shorter hospital stay and improved functional outcomes compared to laparoscopic surgery [9].

These advantages provided by the robotic platform hold the potential to provide improved clinical outcomes [10,11,12].

Both of the mentioned procedures have been compared retrospectively to open and conventional laparoscopic surgery in more trials showing that these methods are safe and feasible [8,13]. However direct comparison of these two techniques are still lacking.

### Potential benefits of robotic rectal resections

#### Technical Benefits

The potential benefits of robotic systems over laparoscopic TME include superior three-dimensional vision, seven degrees of freedom of movement replicating the surgeon’s hand movements made outside the field of the operation, elimination of tremor and superior ergonomics, suggesting possible application in both routine and more difficult cases.

#### Operator Learning Benefits

The main criticisms of robotic TME are the longer operating times and higher costs, compared to laparoscopic TME [13]. More widespread use of and familiarity with the robotic system has led to significant improvements in operation time, rates of conversion to open surgery and the number of harvested lymph nodes.

Bokhari et al. [14] suggested that the learning curve of robotic colorectal surgery was passed at 15 to 25. Kim et al. showed that the learning curve plateaued after 65 cases for Laparoscopic TME as opposed to 32 cases for Robotic TME [15].

Most studies to date about RLAR were done using the da Vinci Si system or older models. With the newer systems X or Xi, and once the surgeons have achieved at least 60 procedures we believe that the outcomes and operating time is less, that has been recently published by the ROLAR trial [11]. Similarly; to achieve good clinical and oncological outcomes with TaTME, surgeons are required to have performed at least 60 cases. Hence we decided to choose 60 procedures as an arbitrary number to select our surgeons experience involved in this trial.

### Potential benefits of TaTME rectal resections

TaTME offers better access to the lower rectum in a difficult pelvis and also facilitate a clear distal margin by insertion of a distal to the tumour purse string under direct vision. This enables an end-to-end anastomosis without the need of multiple stapler firings. This approach also allows two-team synergy with an abdominal and perineal surgeon operating simultaneously.

The technique however still requires significant surgical skills and the ability to operate in a confined space.

The objective of this study is to compare TaTME and RLAR in expert hands. Outcome measures are mainly focused on differences in oncological long-term effects, see ***Table 1*** for details.

**Table 1:** Trial specific primary and secondary outcome measures.


OBJECTIVES	OUTCOME MEASURES	TIME POINT(S) OF EVALUATION OF THIS OUTCOME MEASURE (IF APPLICABLE)

**Primary Objective** 3 year disease free survival after RLAR and TaTME	3 year disease free survival	6 monthly post op for the first 2 years and 12-monthly follow-up for next 3 years

**Secondary/exploratory Objectives** Surgical morbidity/mortality up to 90 days		90 days

Pathological quality assessment	Pathological assessment of mesorectum: Distal resected margin ≥1 cm Lymph node yield Mesorectal plane of surgery R0 resection (all margins clear)	

Assessment of intraoperative adverse events	To report “near misses” and associated impact upon clinical outcomes	

Overall survival at 3 years		

Health economics assessments	Healthcare resource utilization (costs) including hospital length of stay, ICU hours, and productivity losses collected through a Labour Force survey). Return to work/activity will also be included	

Evaluation of the operative length of time	Total OR utilisation time and operative time skin to skin, minutes	

Recruitment per month per centre for the RLAR and TaTME arms	Patients dropout rate during follow-up will also be monitored	


## Methods/design

### Design

**Figure 1:**
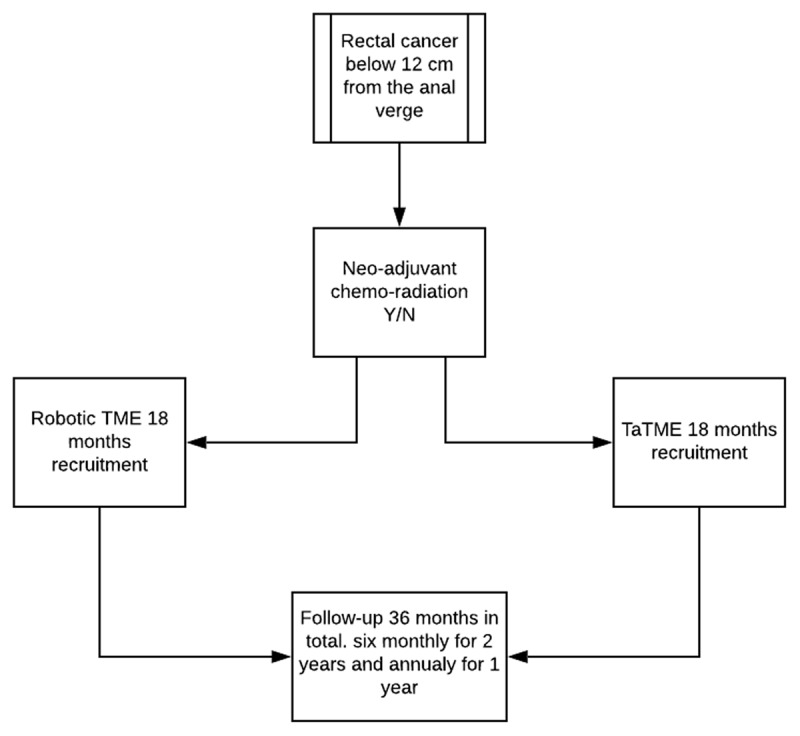
ROTA Study flow.

This is an observational prospective, multicentre trial. Patients will be assigned either RLAR or TaTME depends on the chosen centres. The centres do either TaTME or RLAR for rectal cancer management according to their training and experience. The study will include 330 patients from a number of international sites. This study has no impact on staging investigations, timing of surgery or any other aspect of the patients’ pre, peri or post-operative care. All decisions remain with the local clinician multi-disciplinary team including when to discharge from hospital. However, the biostatisticians performing the analysis will be blinded to the intervention. For study flow see ***Figure 1***.

### Study population

Participants with rectal cancer who are scheduled for initial treatment by TME by the MDT. The inclusion and exclusion criteria are shown in ***Table 2***.

**Table 2:** Trial specific inclusion and exclusion criteria.


INCLUSION CRITERIA	EXCLUSION CRITERIA

Participant is willing and able to give informed consent for participation in the trial.	Female participant who is pregnant, lactating or planning pregnancy during the course of the trial.

Male or Female, aged 18 years or older.	Participant with life expectancy of less than 6 months.

TME surgery for rectal cancer after MDT discussion.	Any other significant disease or disorder which, in the opinion of the Investigator, may either put the participants at risk because of participation in the trial, or may influence the result of the trial, or the participant’s ability to participate in the trial.

Participant has no contra-indication to pelvic radiotherapy at the time of enrolment.	Participants who have participated in another research trial involving an investigational product in the past 12 weeks.

In the Investigator’s opinion, is able and willing to comply with all trial requirements.	

Tumour distance from anal verge – 12 cm or less	

Willing to allow his or her General Practitioner and consultant, if appropriate, to be notified of participation in the trial.	


### The Number of Participants

The primary study end point, DFS is defined as the time following successful surgery to the first date of local, regional/distant relapse or death due to colorectal cancer (patients with secondary malignancy will be censored). Overall survival is defined as the time following successful surgery to death.

In the TaTME, it is assumed that the DFS is 92% and in the RLAR arm 95% at 3 years. A non-inferiority margin of 90% is selected. Based on this estimation, sample size calculation has been done with a one-sided level of significance of 20% and a power of 80%. A total number of 330 patients is needed, 165 patients in the RLAR arm and 165 patients in the TaTME are required with a 10% loss to follow-up assumed.

DFS will be analysed on an intent-to-treat (per protocol) basis using a log-rank test after propensity matching analysis has been conducted; a secondary analysis of DFS will also performed be using a Cox proportional hazards regression model with the unmatched cohort (without adjustment of baseline characteristics, i.e. propensity score match) that allowed for the effect of treatment and include lists of covariate with a statistical interaction of alpha level of 20%.

### Data Management

Case report forms (CRFs) will be entered in a secured online page. Only authorized staff at sites will have access via an individual secure login username and password to enter the data. All paper CRFs must be completed, signed/dated and returned to the investigator. Data reported on each CRF should be consistent with the source data or discrepancies should be explained. If information is not known, this must be clearly indicated on the CRF. All missing or ambiguous data will be queried. All sections are to be completed.

All trial records will be archived and securely retained for at least 25 years.

### Ethics and dissemination

The trial will be performed in accordance with the recommendations guiding physicians in biomedical research involving human subjects, adopted by the 18^th^ World Medical Association General Assembly, Helsinki, Finland and stated in the respective participating countries laws governing human research, and Good clinical Practice. The medical ethical committees of all participating countries will have to approve the study protocol prior to enrolling patients. The protocol has been approved by the Danish ethical committee of the Region of Southern Denmark with reference number S-20210038.

## Discussion

To date studies on TaTME and robotic TME have focused mainly on short term outcomes and oncological endpoints such as specimen quality, circumferential resection margin involvement and the free distal margin [13,16,17].

In a recent study comparing TaTME and Robotic TME, there were no differences in the incidence of poor quality. However the authors noted that distal resection margin (DRM) involvement was higher in the TaTME group [16]. They attributed this to the learning curve effect of TaTME. As discussed earlier, previous reports have noted that the learning curve for robotic TME was less than laparoscopic surgery, which in effect TaTME is still a laparoscopic “bottom up” approach.

Nonetheless, the long-term oncological parameters for both procedures, including overall survival, disease-free survival and local recurrence, are yet to be clarified. Secondly, there have been significant concerns in regards to an increased recurrence rate after TaTME [18]. The Norwegian group have noted an unexpected increase in local recurrence after TaTME of 9.5% after a median of 11 months after surgery [18]. The reasons for their observations is unclear, despite that fact that their surgeons performing TaTME were experienced and were proctored and trained in England and Spain for the procedure.

In 1980 Knight and Griffen [19] publised their stapling technique for low rectal cancer anastomosis using linear and circular stapler. This has led to the introduction of the double stapling technique [20]. The advantage is that the distal segment of the bowel is not opened and this avoids spillage from the rectal stump.

The possibility of increased local recurrences in TaTME may be related to the rectal transection and air flow during dissection from the perineum. The rectal purse-string suture is never completely air tight and this will invariably lead to shredding of microscopic cancer cells in the pelvis and hence this may lead to an increase rate of local recurrence. During a robotic TME and the use of the articulating robotic stapler, the rectal transection is precise and there are no leakage of microscopic cancer cells in the pelvis.

With these current issues in mind, we aim to compare the 3 year disease free survival and local recurrence rate of the two procedures performed by experienced surgeons who are beyond their learning curve. Only surgeons who have performed more than 60 procedures in either TaTME or robotic TME will be selected.

ROTA study will be the first multicentre prospective trial comparing RLAR and TaTME in regards to disease free survival (DFS) and other clinical trials.

## Conclusions

There is an urgent need to assess and compare the long-term oncological outcomes of newer surgical techniques for TME (TaTME & RLAR). This will allow recommendations to be made for more tailor made treatment of rectal cancer, based on detailed outcomes from expert centers.

## Figures and Tables

**Table:** 

TME:	Total Mesorectal Excision
TaTME:	Transanal assisted Total Mesorectal Excision
RLAR:	Robotic assisted Low Anterior Resection
RC:	Rectal Cancer
DFS:	Disease Free Survival
MDT:	Multi Disciplinary Team
ICU:	Intensive Care Unit
OR:	Operating Room
TAMIS:	Trans anal Minimally Invasive Surgery
CRM:	Circumferential Resection Margin
BMI:	Body Mass Index
RCT:	Randomized Controlled Trial
DRM:	Distal Resection Margin
CRF:	Case Report Form
